# Identification of Bacteria Synthesizing Ribosomal RNA in Response to Uranium Addition During Biostimulation at the Rifle, CO Integrated Field Research Site

**DOI:** 10.1371/journal.pone.0137270

**Published:** 2015-09-18

**Authors:** Lora R. McGuinness, Michael J. Wilkins, Kenneth H. Williams, Philip E. Long, Lee J. Kerkhof

**Affiliations:** 1 Department of Marine and Coastal Science, Rutgers University, New Brunswick, NJ, United States of America; 2 School of Earth Sciences, Ohio State University, Columbus, OH, United States of America; 3 Earth Sciences Division, Lawrence Berkeley National Laboratory, Berkeley, CA, United States of America; Argonne National Laboratory, UNITED STATES

## Abstract

Understanding which organisms are capable of reducing uranium at historically contaminated sites provides crucial information needed to evaluate treatment options and outcomes. One approach is determination of the bacteria which directly respond to uranium addition. In this study, uranium amendments were made to groundwater samples from a site of ongoing biostimulation with acetate. The active microbes in the planktonic phase were deduced by monitoring ribosomes production via RT-PCR. The results indicated several microorganisms were synthesizing ribosomes in proportion with uranium amendment up to 2 μM. Concentrations of U (VI) >2 μM were generally found to inhibit ribosome synthesis. Two active bacteria responding to uranium addition in the field were close relatives of *Desulfobacter postgateii* and *Geobacter bemidjiensis*. Since RNA content often increases with growth rate, our findings suggest it is possible to rapidly elucidate active bacteria responding to the addition of uranium in field samples and provides a more targeted approach to stimulate specific populations to enhance radionuclide reduction in contaminated sites.

## Introduction

The long history of mining uranium and other toxic chemicals needed for the production of nuclear weapons has left numerous environmental problems for the US Dept. of Energy. At the Integrated Field Research Challenge site in Rifle Colorado (IFRC), vanadium and uranium were mined for 34 years to develop the US nuclear arsenal and the tailings were stored on site, contaminating the subsurface. Measurements of uranium in groundwater have detected elevated concentrations up to 1.5 μM, which is above the EPA limits for safe drinking water. This uranium in the Rifle groundwater is predominantly in the soluble U(VI) form, which becomes virtually insoluble when reduced to the U(IV) form. Prior research at the site has demonstrated acetate biostimulation leads to a decrease in uranium groundwater concentrations, below the safe drinking water limits [1, 2]. Concurrent with the U (VI) removal, shifts in the microbial community have indicated an increase in *Geobacter*-like microorganisms [3]. Additional efforts to determine the *in-situ* populations during the biostimulation experiments have included proteomics analysis of the *Geobacter*-like targeted peptides [4], clonal libraries of 16S rRNA genes of sediment and groundwater samples [5] and stable isotope probing (SIP) using ^13^C-labeled acetate [2, 6, 7]. Unfortunately, elucidation of the microbial populations which are potentially responsible for the bioreduction of uranium at the Rifle site has been not accomplished.

This difficulty results from the problems in assigning a role to a particular microorganism for dissimilatory or detoxification processes within an environmental sample. Rather, a less direct approach of assessing a physiological response (e.g. ATP production, ribosome synthesis, protein synthesis, etc.) from U (VI) additions becomes necessary. This activity measure with U (VI) addition should discern those microorganisms involved in U (VI) respiration from those bacteria passively interacting with uranium in the stationary phase/spores and from those bacteria which do not interact with U (VI) at all. Of these physiological responses, only ribosome and protein synthesis have the potential to differentiate various active microorganisms. However, a specific functional protein associated with uranium reduction remains elusive with c-type cytochromes, pili/nanowires, and extra cellular electron carriers all playing a role among various microorganisms [8].

Therefore, this study was initiated to assess if bacteria actively responding to U (VI) addition could be detected in several IFRC wells treated with acetate during 2008 and 2009 (e.g.[2]) by characterizing ribosome production. The concept is predicated on monitoring the bacterial rRNA response by terminal restriction fragment polymorphism analysis (RT-TRFLP). RNA content has been shown to be related to growth rate of bacteria under a broad range of conditions [9–13]. Furthermore, the RT-TRFLP profiling of ribosomes has been used to target another dissimilatory process (dehalogenation) to identify a microorganism capable of debromination [10]. For this study, Rifle groundwater samples were collected shortly after field uranium concentrations declined to their lowest levels following acetate addition. It was assumed that amendments of U (VI) and acetate to these groundwater samples would stimulate those bacteria that have recently engaged in uranium respiration or might be able to use uranium as an electron shuttle. These microorganisms will likely be the only bacteria with the necessary cellular machinery to respond quickly to changes in U (VI) concentrations in a dose dependent manner by synthesizing ribosomes. Our short term incubations (24 h) and rRNA/TRFLP analysis indicated 4 different bacterial OTUs were synthesizing rRNA in response to increasing U (VI) amendment. These microbes are closely related to iron or sulfate-reducing bacteria. Identifying the bacteria directly responding to U (VI) additions at Rifle can lead to a more targeted biostimulation approach for the specific microorganisms reducing radionuclides at contaminated sites.

## Materials and Methods

### Field Site

**Fig 1:**
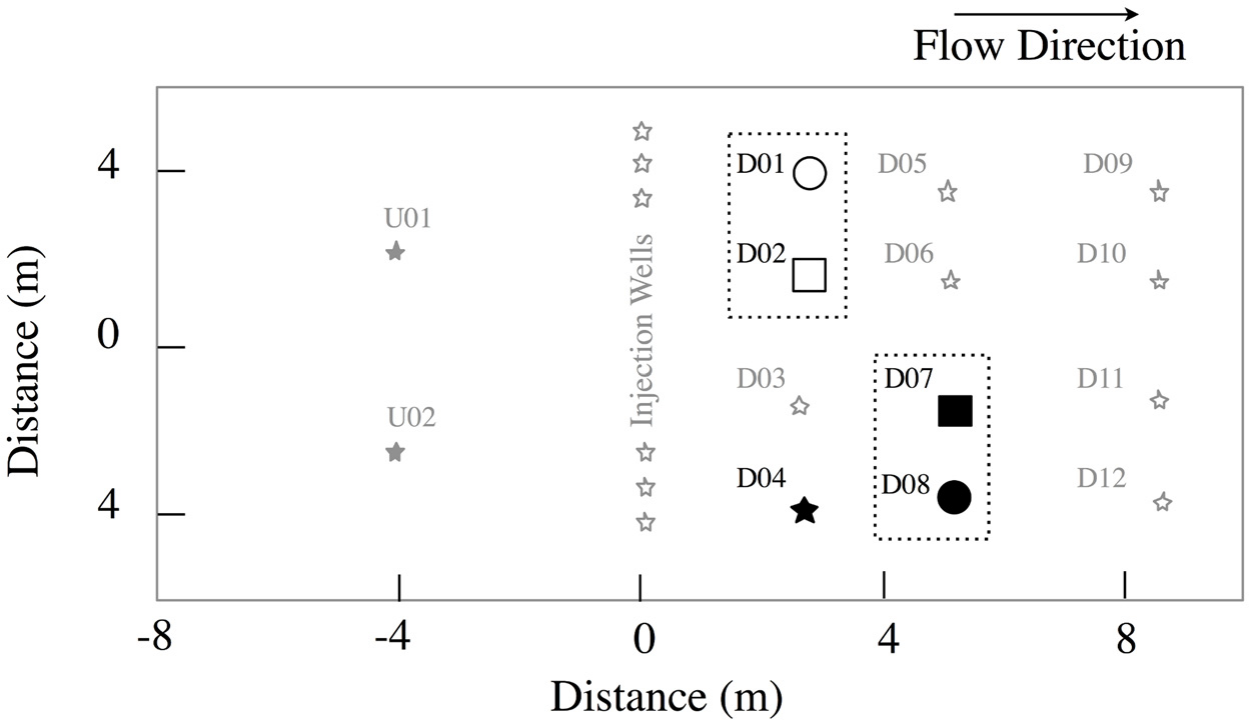
Map of the field site indicating the wells used for this study.

This research was done at the Integrated Field Research Challenge Site (IFRC) at Rifle which is owned by the City of Rifle, CO. DOE's Office of Legacy Management and its associated researchers are provided access to the site by the City of Rifle through a letter of agreement until 2018. (Additional access for conducting scientific research can be obtained by contacting Dr. Kenneth Williams at Lawrence Berkeley National Laboratory.) A detailed description of the site is found in Williams et al., [2]. During the 2008 and 2009 field experiments, subsurface chemical concentrations were monitored using the following methods: acetate/bromide by ion chromatographic analysis (Dionex ICS-1000), soluble uranium by kinetic phosphorescence analysis (Chemchek Instruments, Richland, WA), Fe(II) by the phenanthroline-II method, and sulfide using methylene blue [2]. In addition to the uranium microcosm study reported below, 500 L groundwater samples were collected during the 2008 field amendment from well D04 for proteomic research by filtering onto 1.4 μm pre-filter membranes (and ultimately 0.2 micron filters) using a tangential flow system (Fig 1; [4]). The DNA extracted from these pre-filters was amplified with the primers 27 Forward (labeled with 6-FAM) and 519 Reverse and profiled by TRFLP as described previously [2] to monitor microbial population dynamics within the subsurface during the field experiment.

### Uranyl Sulfate Amended Microcosms Studies

Groundwater from wells D02 and D07 were used for the uranium microcosms in 2008 and from wells D01 and D08 in 2009. For collecting the groundwater microbial community, each well was purged (20 liter volume) and 120 ml serum bottles were allowed to overflow 4 times before being sealed and capped. Additions of 1 mM acetate and uranyl sulfate (0–8.0 μM in 2008/22 days-post initiation of acetate injection); 0–4 μM in 2009/19 days-post initiation of acetate injection) were the experimental treatments for determining the uranium responsive bacteria. Control incubations included no acetate, acetate- no uranium, and acetate + sulfate (2 μM)-no uranium treatments. The bottles were incubated for 24 hours in the dark in insulated containers immersed in groundwater to maintain ambient temperatures. After incubation, the biomass was collected on a 0.2 μm filter (Supor, Pall Corporation, NY). The filter was flash frozen in liquid nitrogen in the field and stored until shipment to the laboratory by the use of a dry shipper. Filters were placed at –80°C until extraction in the laboratory.

### RNA/DNA Purification

Total nucleic acids (DNA and RNA) were purified from the filtered samples using a phenol/chloroform/isoamyl alcohol extraction protocol [14]. The nucleic acids were precipitated with ethanol, and re-suspended in DEPC treated water. Care was taken to ensure nucleic acid pellets from all samples were re-suspended in the same volume and all subsequent steps preserved the initial RNA concentrations. RNA amplifications were performed by first digesting the DNA in the sample with Turbo DNA-free^TM^ kit (Life technologies; Grand Island, NY) at 37°C for 20 minutes. The sample was then diluted 10^−4^ to maintain appropriate template concentrations for the RT-PCR reaction to minimize PCR bias from excessive target molecule concentration [10]. The diluted rRNA samples were amplified using the Titan One Tube RT-PCR kit (Roche Applied Science, IN) with 16S rRNA universal primers 27 Forward (5' AGA GTT TGA TCC TGG CTC AG 3'; fluorescently labeled with 6-FAM) and 519 Reverse (5' ATT ACC GCG GCT GCT GG 3'). Amplification parameters were 1 cycle at 50°C for reverse transcription at 30 minute, followed by 23 cycles of 94°C for 30 seconds, 57°C for 30 seconds and 72°C for 1 minute with a final extension time of 7 minutes. No-RT controls were performed on all samples to assure only RNA was being amplified and profiled. No amplified products were observed in any of the no-RT controls.

### RT-TRFLP Quantitation and Replicability Analysis

Two microliters of RT-PCR product was digested with *Mnl I* endonuclease (New England Biolab, Beverly, MA). All digests were in 20 μl volumes for 6 h at 37°C. Sodium acetate and glycogen were added to the digestion reaction and 37 μl of 95% ethanol to precipitate the DNA [14]. The samples were dried, then re-suspended in 19.7 μl de-ionized formamide and 0.3 μl ROX 500 size standard (Applied Biosystems). TRFLP fingerprinting was carried out on an ABI 310 Genetic Analyzer (Applied Biosystems, Foster City, CA) using Genescan software and an internal size standard. Peak detection was set at 25 arbitrary fluorescent units and the area was determined by the Genescan software.

To verify the RT-TRFLP procedure yielded a quantitative response to input rRNA concentration, triplicate samples containing varying mixtures of ribosomes from *Escherichia coli*, *Vibrio fisherii*, and well D01 were analyzed. A cocktail of rRNA was prepared by first diluting each RNA sample to similar 16S rRNA concentrations based on ethidium bromide fluorescence in agarose gels [15]. Then RT-TRFLP test cocktails were created by mixing the RNA samples at various dilutions by volume (1:1:1, 1: 0.2:0.05, and 0.2:1: 0.2 of template respectively). These test cocktails were further diluted by 10^−4^, amplified, and analyzed by RT-TRFLP as described above. The peak area for *E*. *coli*, *V*. *fisherii*, and the dominant rRNA peaks from D01 (TRF 212 and 214) in the 1:1:1 mixture were then used to calculate/predict the peak area for the 1:0.2:0.05, and 0.2:1: 0.2 mixtures. The results of the predicted areas vs. the actual areas measured for the mixtures are shown in the supplemental material (S1 Fig; n = 8 TRF peaks). Triplicate analysis of the RNA mixtures demonstrated good reproducibility and indicated that the method could provide a predictive measure of the change in relative abundance in the original target molecule concentration. Specifically, a higher template concentration resulted in a proportionally larger total TRF peak area in the profile. Only at very high target concentrations for a single sample were the predicted values lower than the measured values by RT-TRFLP.

### Sequence Identification of RT-TRFLP Peaks

Clonal libraries of RT amplicons were constructed from wells D01, D02, D07, and D08 plus well D04 (day 22) using DNA-based 16S rRNA gene amplicons (500 bp from 27F to 519R) and the Topo TA cloning kit, as per the manufacturer’s instruction (Invitrogen, CA). Individual colonies were then screened by a multiplex format [16] to determine the TRF of the recombinant amplicons. The inserts that matched peaks from the RT-TRFLP profiles were sequenced using M13 primers (Genewiz, Inc. NJ). The sequences were compared to known sequences using BLAST and a maximum likelihood phylogenetic tree was re-constructed using 438 unambiguously aligned bases with Geneious v. 4.5 analysis software [17, 18].

## Results

### Field Site and Uranium Concentrations in Groundwater

**Fig 2:**
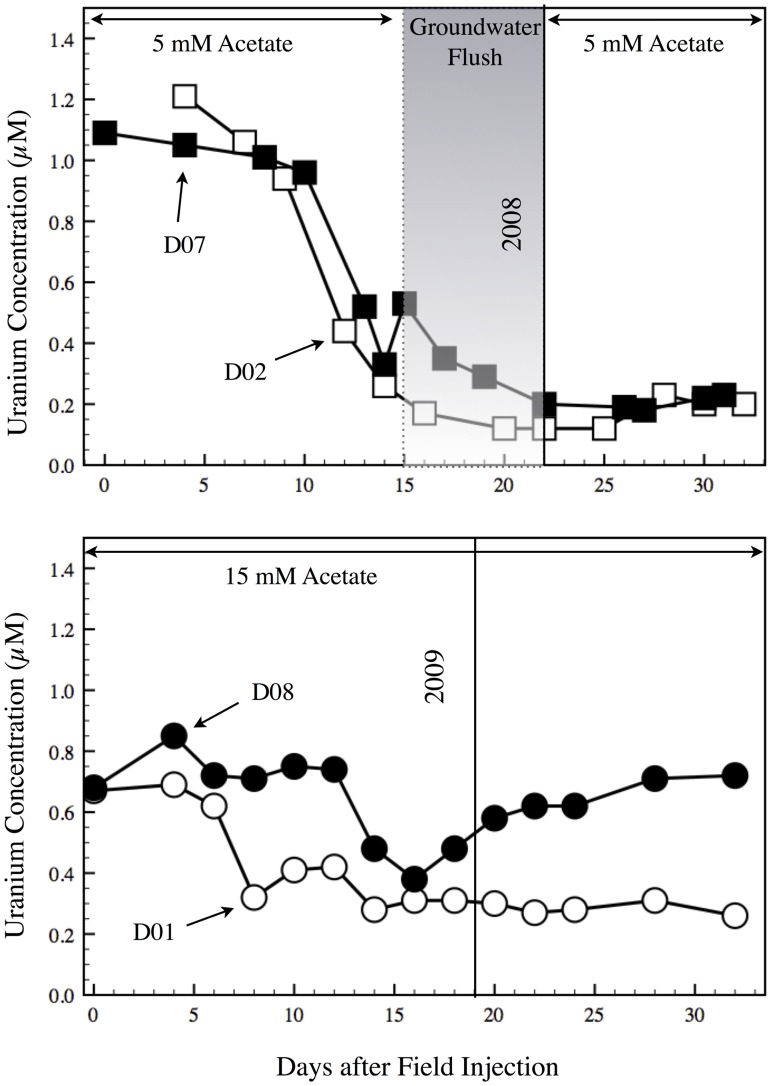
Uranium concentrations from groundwater collected from wells D02 (open square), D07 (closed square); 2008 and D01 (open circle), D08 (closed circle); 2009 Vertical lines indicate the time after field acetate injection when the various wells were sampled for the uranium amendment studies. The groundwater flush in 2008 is indicated in grey shading.

The Rifle IFRC experimental plot A consists of a gallery of injection/monitoring wells that were installed in 2007 (Fig 1). A detailed description of the site is found in Williams et al., [2]. In the 2008 field experiment, the acetate amendment (with bromide as a conservative tracer) was initially targeted at 5 mM (Day 0–15), separated by a groundwater flush to monitor the bacterial community’s return to pre-acetate amendment conditions (Day 15–24), which was followed by 5 mM acetate addition (Day 24–38; Fig 2). For the 2009 field experiment, acetate was added continuously at 15 mM for 30 days. Field amendment of acetate lowered the concentration of uranium in groundwater from 0.7–1.2 μM to 0.2–0.4 μM within 20 days for all monitoring wells used in this study (Fig 2). The well gallery demonstrated comparable loss kinetics of soluble uranium in 2008 and 2009 upon acetate addition.

At the time of the 2008 collection, the concentrations of uranium for the D02 well (Fig 2; open square) showed a rapid decrease followed by a stable, lower concentration during and after the groundwater flush for the next 15 days. Similarly, D07 (Fig 2; closed square) displayed a comparable drop in uranium concentrations with a small spike in concentration at the onset of the groundwater flush. Interestingly, a slightly different pattern was observed in the 2009 field amendment, which lacked a groundwater flush and had a 3x higher acetate amendment. In well D01 (Fig 2; open circle) the uranium concentration was approximately 30% lower at the onset of the acetate field amendment, dropping quickly and remaining low for the next 25 days. However, for well D08, uranium concentration initially dropped to comparable levels as well D02 (Fig 2; closed circle) during the first half of the field amendment and began to rise thereafter. This change in uranium concentration at D08 probably reflected subsurface alterations in flow patterns and acetate delivery in 2009.

### Uranium Incubations

The intent of the experimental design is that the addition of uranium sulfate (and acetate) to groundwater samples depleted in soluble uranium should stimulate only those bacteria capable of respiring uranium or using uranium as an electron shuttle at the time of sampling. Any increase in U (VI) respiration or the transfer of electrons to alternate terminal electron acceptors will likely lead to the production of ATP, the synthesis of ribosomes, and growth in the microorganisms responding to uranium amendments. Ribosome synthesis has been shown to be tightly correlated to growth rate in a number of *Proteobacteria* [8, 11, 12]. Therefore, groundwater samples for the microcosms were collected close to the lowest uranium concentrations, ranging from 0.2 to 0.5 μM uranium (Fig 2) from monitoring wells (D01, D02, D07, and D08) during the 2008 and 2009 acetate field-amendment.

The control incubations were extremely important for assessing if changes in RNA content could be attributed to the addition of uranyl sulfate. The acetate-no uranium incubations were established to determine the level of RNA template resulting from electron donor alone. In our study, the production of rRNA from acetate alone was either negligible or non-detectable for all samples (S2B and S3 Figs). Likewise, the control incubations of acetate + sulfate at 2 μM concentrations did not stimulate rRNA synthesis in our microcosms, implying growth on sulfate was too slow to be detected by ribosome synthesis during our 24 hour incubation, even in the presence of 0.5 micromolar uranium (S3 Fig). The lack of response with sulfate addition was not surprising given that the groundwater sulfate concentrations at the site ranged from approximately 3–10 mM. Therefore, addition of micromolar amounts of sulfate from our uranyl sulfate, acetate + sulfate, and the sulfate-only controls would not appreciably alter ambient concentrations.

**Fig 3:**
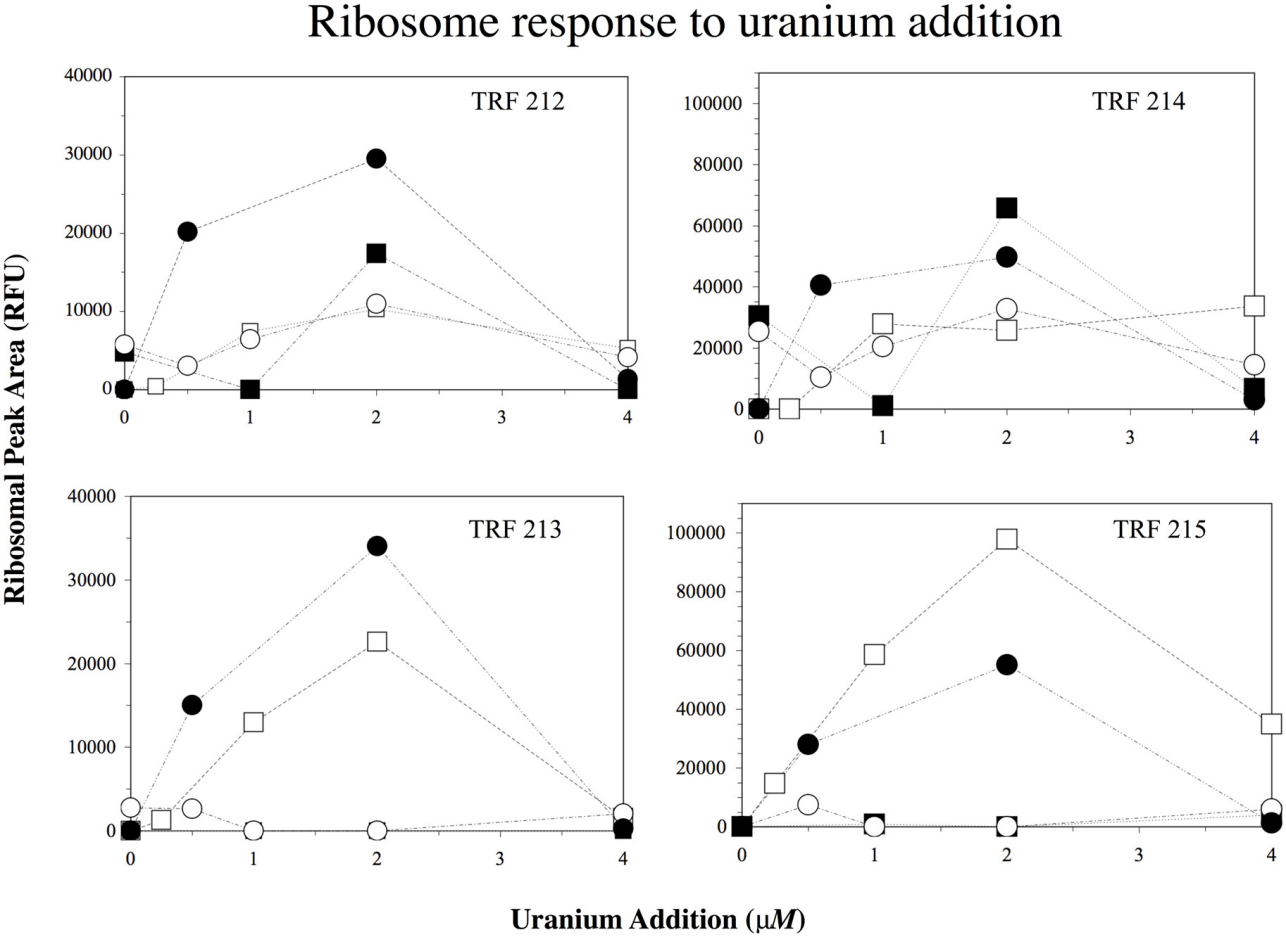
Ribosomal response to uranium additions in groundwater after 24h for 4 OTUs (TRF-212, 213, 214, 215 bp) from wells D02 (open square), D07 (closed square), sampled in 2008, D01 (open circle), D08 (closed circle), sampled in 2009 (note the difference in scales).

The results from increasing uranyl sulfate addition on the RT-PCR profiles from biological replicates during subsequent years are presented in Fig 3. Only a few TRFs were found to comprise the majority of any individual RT-PCR community profile from the various samples, due to the high dilution factor of RNA before amplification (10^−4^; TRFs-212, 213, 214 and 215 using *Mnl I*). Each of these 4 TRF’s accounted for 5–70% of the overall RNA profile sample from any given microcosm experiment. The 215 TRF displayed the highest RT-PCR fluorescent area from the uranium additions to groundwater microcosms, with the 214 TRF being approximately 50% of the highest 215 signal and 212, 213 being roughly 33% of the highest 215 signal. Each TRF displayed a slightly different response to uranyl sulfate addition for the individual sampling wells. For example, in well D08 (closed circle) all TRFs exhibited a dose dependent increase in rRNA signal to changes in uranium concentration from 0 to 2 μM. At 4 μM the rRNA signal returned to no-amendment control (Fig 3). A comparable low rRNA signal (similar to the no amendment control) was observed with additions up to 8 μM. For well D02 (open square), all TRF (except 214) also displayed the same dose-dependent response. In well D02, the overall RNA signals from TRFs 212 and 213 were 2x lower than D08 while the TRF 215 RNA signal was 2x higher. Interestingly, the TRF 214 RNA signal at well D02 increased with uranium addition and was actually highest at 4 μM (Fig 3). For well D01 (open circle), only TRF 212 demonstrated a dose dependent response, while TRF 214 was more variable. TRF 213 and 215 did not show any change in RNA signal with increasing uranium concentration. Well D07 had the lowest RNA response to uranium addition for all TRFs. TRF 212 and 214 actually showed a loss in RNA signal with 1 μM doses of uranium compared to the no-uranium controls. However, the 2 μM uranium addition led to an increase in RNA signal. TRF 213 and 215 did not demonstrate any increase in RNA for well D07 at any uranium addition.

**Fig 4:**
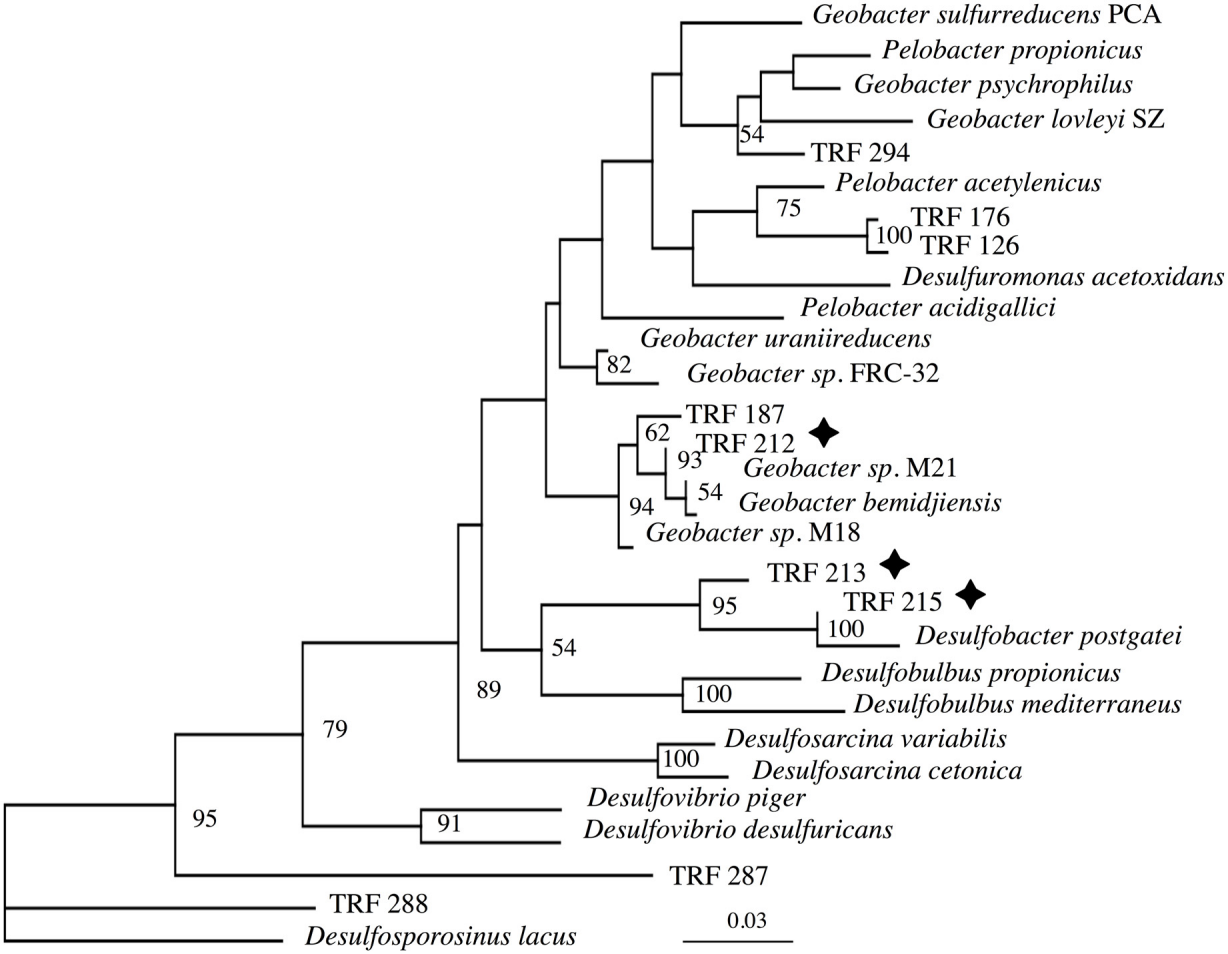
Phylogenetic tree of active TRF’s resulting from the uranium additions (star) are shown with the nearest cultured relatives. The reconstruction was done using maximum likelihood methods on 438 aligned bases. Bootstrap values >50 for 100 iterations are indicated.

In order to identify the microorganisms associated with the various TRFs, clone library analysis of the RT-PCR products was performed. Individual clones were screened by TRFLP to match the 500 bp inserts with specific TRFs. Sequence of these 500 bp SSU gene fragments indicated TRFs 215 and 213 were related to *Desulfobacter postgateii* and TRF 212 was identified as closely related to *Geobacter bemidjiensis* (Fig 4). The similarity of the *Desulfobacter*-like clones ranged from 94–99% and the similarity of the *Geobacter*-like clone was 98%. TRF 214 was not recovered from the RT-PCR amplicon library after screening over 60 clones.

### Groundwater Samples During the 2008 Field Addition of Acetate

**Fig 5:**
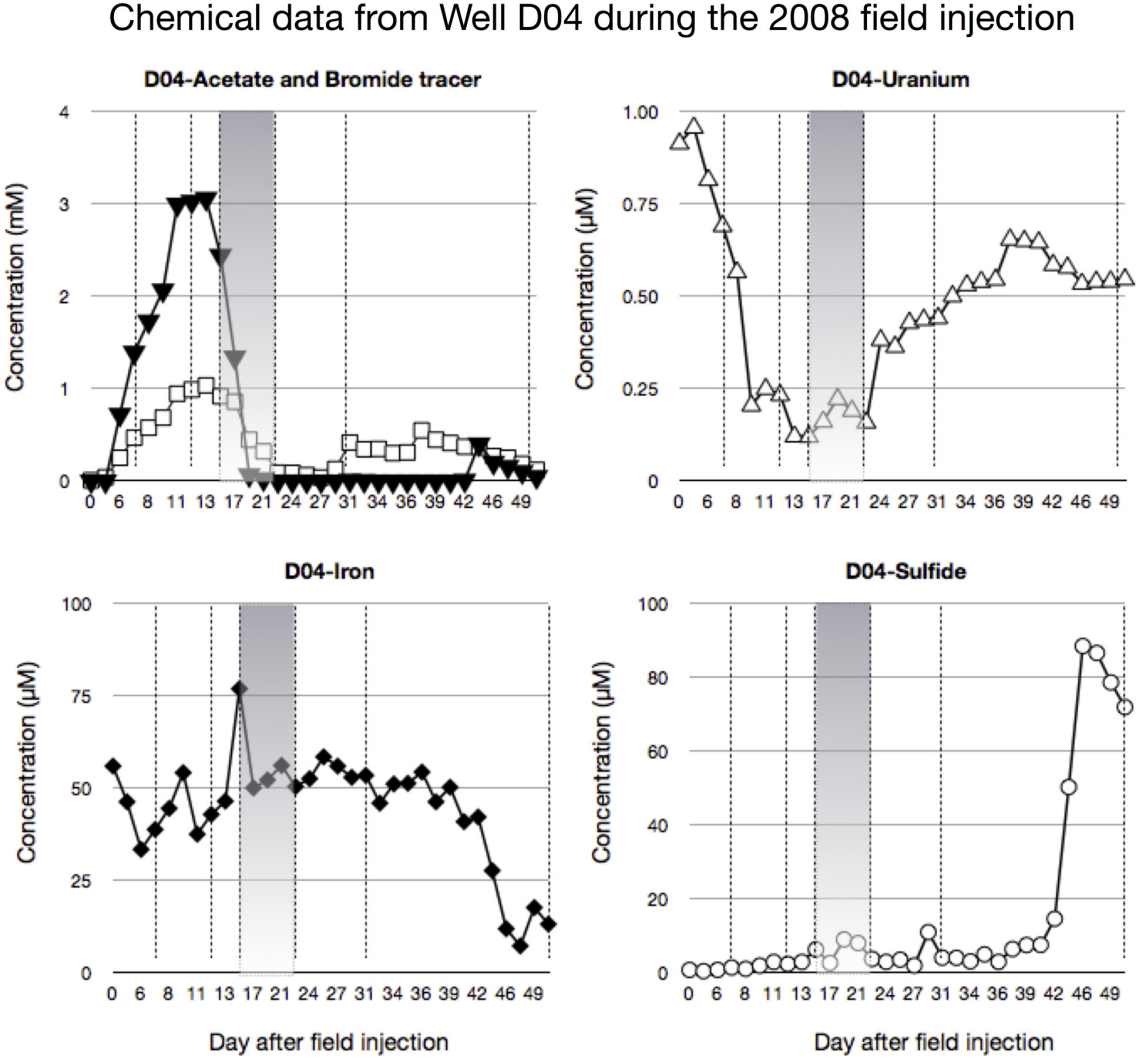
Chemical analysis of groundwater from well D04 during the 2008 field experiment-acetate (closed triangles) and bromide (open squares). The period of groundwater flush (shaded) and the times of biomass sampling (dotted line) are indicated. The chemical methods used in this analysis exhibit analytical variability in the femto-, nano-, and micro-molar range for uranium, iron/sulfide, and acetate respectively. All field measurements are beyond this range.

To ascertain whether the same microorganisms that were observed making ribosomes in the uranium microcosms were also stimulated during the 2008 field amendments with acetate, 16S rRNA gene analysis from genomic DNA was performed on groundwater samples from well D04. Chemical analysis of the groundwater during this experiment with the dates of biomass collection (dotted lines) and the groundwater flush (shaded grey) is presented in Fig 5. Increases in D04 acetate and bromide can be seen during the first 12 days of field treatment with a subsequent loss in soluble uranium. This time frame corresponds with the highest peak in soluble iron concentration from 50 to 75 μM. During the groundwater flush, acetate/bromide and Fe (II) concentrations decreased while uranium and sulfide show a slight increase then a return to pre-flush concentrations. After the groundwater flush, there is a extended period with no increase in acetate/bromide and a gradual increase in uranium (Day 22-Day 30), suggesting the field amendment is not reaching well D04 due to changes in groundwater flow. After day 30, there is a step-wise increase in bromide, while acetate remains undetectable until day 45. The iron concentration decreases while the sulfide concentration increases during the final 10 days of treatment.

**Fig 6:**
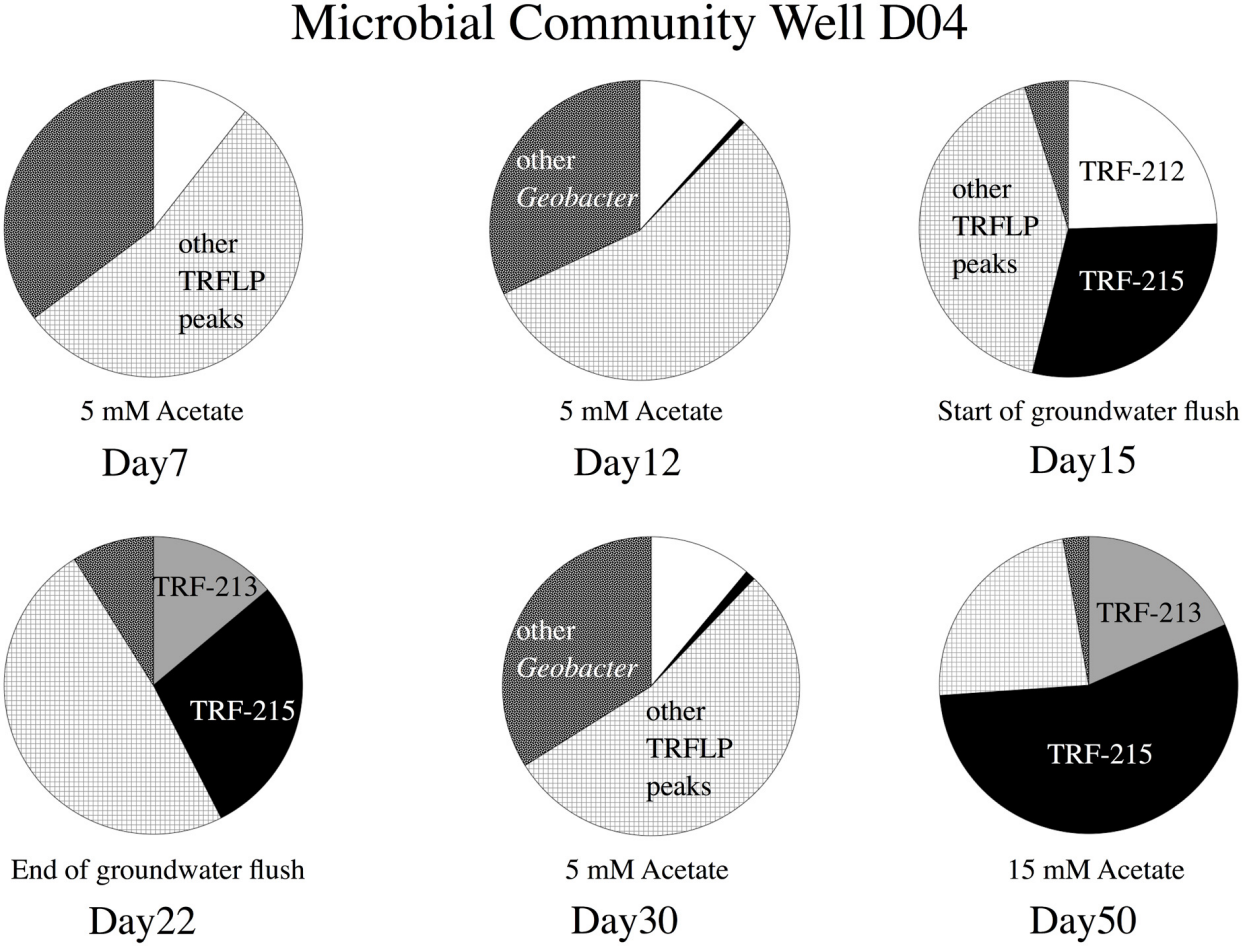
Percent total peak area from community DNA of 16S rRNA genes TRFLP profiles of pre-filters 2008 well D04 during field acetate injection: TRF 212-white, 213-light grey, 215-black, other *Geobacter*-like TRF’s-dark grey stipple, all other TRF peaks-light checked.

During this 2008 field amendment, 6 biomass samples were collected on 1.4 micron pre-filters and tangential flow membranes for proteomics [4]. These pre-filters routinely became clogged during the field sampling due to the large volumes of water being collected (500 L), effectively reducing the nominal pore size significantly. The groundwater bacterial community captured on the pre-filters was characterized by DNA-TRFLP as described above to determine if the same TRFs growing on U (VI) in the micrcosms are responding to the acetate injections in the subsurface (S4 Fig). The results indicated a relatively stable bacterial community profile following acetate amendment on Day 7 and Day 12 with *Geobacter*-like TRFs (dark grey) and TRF 212 (white) representing 44–46% of the overall profile (Fig 6, see [2] for other *Geobacter*-like TRFs). This corresponds with a saw tooth pattern in soluble iron, a decrease in soluble uranium, and the emergence of TRF 215 (black). By Day 15, a large change in bacterial community is observed. The *Geobacter*-like TRFs (with the exception of 212) are replaced by TRF 215 (30% of the community) with a reduction of the other TRFs. This time frame corresponds with a 1.8 fold increase in the soluble iron concentration, a decrease in the uranium concentration to below 0.122 μM, and a doubling of sulfide from 3 to 6 μM. As the groundwater flush proceeds, there is a large initial drop in iron followed by a transient increase/decrease in uranium, sulfide, and iron at the new level (Fig 5). By Day 22 after the flush, the bacterial community remains largely intact with the exception of TRF 212 which is completely replaced by TRF 213 (light grey; Fig 6). After the flush, acetate/bromide do not re-appear from Day 22 to 30 in well D04 and the community resets to the Day 12 community. By Day 50, the bacterial community has shifted from an iron reducing to a sulfidogenic community with nearly 75% of the profile comprised of the TRFs 213 and 215, which are related to *Desulfobacter*.

## Discussion

The Rifle IFRC was established to help resolve major research questions about the movement of radionuclides and other contaminants in the subsurface. One question is which groups of microorganism are capable of growth on uranium *in situ*. Initial studies demonstrated that *Geobacter-like* species responded to acetate addition at Rifle, while soluble uranium levels decreased [1, 5]. These *Geobacter*-like blooms were generally followed by *Desulfobacter-* and *Desulfovibrio*-like groups increasing as the community shifted from iron to sulfate reduction [19, 20]. The research presented here also indicates comparable responses in rRNA synthesis by *Geobacter-* and *Desulfobacter*-like groups to acetate additions during the 2008 and 2009 field experiment. However, not all iron or sulfate reducers responded in a uniform manner or seem to be associated with uranium reduction.

For example, only one member of the *Geobacter* group (TRF 212) was found to make ribosomes in response to uranium additions, indicating this microorganism may also play a role in uranium reduction at the Rifle site. In addition to this report, this *G*. *bemidjiensis*-like TRF has also been identified several times in Rifle samples, as well as in column incubations done with Rifle sediment and groundwater [2, 21]. Likewise, proteomic-based analysis of active, *in-situ* microbial communities at Rifle in 2007 demonstrated a *G*. *bemidjiensis*-like microorganism as an active member of the subsurface community [4]. Recently, several different *Geobacter* isolates from the Rifle site and the Oak Ridge Field study site in Tennessee have also been shown to reduce uranium using a resting cell assay including *G*. *uraniireducens*, *G*. *daltonii*, and *G*. *sulfurreducens* [22–24]. Unfortunately, these studies did not indicate whether these *Geobacter* isolates were capable of growth on uranium, only that cell suspensions can induce radionuclide reduction at high uranium concentrations.

At the Rifle site, a *Geobacter bemidjiensis*-like microorganism (TRF 212) was shown to make ribosomes in response to uranium amendments. In addition, there were other microbes that when incubated with increasing uranium concentrations, increased their 16S rRNA content in a dose-dependent manner in multiple wells at the Rifle study in subsequent years. Furthermore, these bacteria demonstrated a higher ribosomal RNA response to the additions of uranium as a terminal electron acceptor than the *G*. *bemidjiensis*-like TRF-212. For example, the TRF 215 (closely related to *Desulfobacter postgateii)* had the highest rRNA signal and (presumably) growth rates in the microcosms, and may play an important role in reducing radionuclides at the site. Interestingly *D*. *postgateii* was not found to reduce uranium at 100 μ*M* [25], consistent with our findings that the *D postgateii*-like microorganism at Rifle is sensitive to high uranium concentrations. The uranium concentrations eliciting a toxic response in this study (>2 μM) are also in line with findings of uranyl ion toxicity at 1 μM on the pyrroloquinoline quinone molecule in *Pseudomonads* [26]. Finally, there are suggestions that other sulfate reducers can reduce uranium. Pietzsch *et al*. [27] isolated a *Desulfovibrio* that was able to reduce uranium and grow. *Desulfotomaculum reducens* has been reported to grow on Fe (III), Cr (VI), Mn (IV) and U (VI) [28]. These findings all indicate the scope of available terminal electron acceptors for microorganisms classified as sulfate reducers is quite diverse and assays to determine which microorganisms may be capable of growth on uranium have routinely been conducted at toxic concentrations.

However, it should be noted that in this study the redox state of the uranium was not directly determined within the microcosms to verify cellular respiration. Additionally, our data on rRNA synthesis can not rule out the concept that uranium is potentially acting as an electron shuttle or kinetically stimulating alternate anaerobic respiratory pathways in our microcosms. Interestingly, most descriptions of electron shuttles, such as AQDS or humic acids, indicate these compounds are soluble in both the reduced and oxidized states. For this reason, electron shuttles can accept an electron at the bacterial cell surface, diffuse towards a solid surface (e.g. iron oxides), deposit that electron, and diffuse back to the cell surface to receive another electron. In contrast, uranium is highly insoluble when reduced. This change in solubility greatly diminishes the ability for uranium to diffuse toward a solid surface or a soluble compound and should suppress electron shuttling. Although there is a report of U shuttling of electrons to Fe (III) under very different experimental conditions [29], the groundwater microcosms in this study did not have any detectable *Shewenella* species, did not contain iron oxides clays at 2 g/L, did not contain 5 mM lactate, contained 2 μM uranyl sulfate rather than 830 μM uranyl acetate, and were incubated for 24 h rather than 240–340 h. Furthermore, it is unclear why uranium (acting as an electron shuttle) would only be effective over a very narrow concentration range in stimulating rRNA synthesis. Presumably, the presence of excess electron shuttle would not suppress rRNA synthesis at higher concentrations. Another possible mechanism to account for our rRNA synthesis data is that the uranium amendment in our microcosms is kinetically stimulating sulfate reduction by acting as a catalyst. Again,it is unclear by which catalytic mechanism this uranium would only stimulate sulfate reduction at a concentration of 0.5 to 4.0 micromolar, yet suppress sulfate respiration (and rRNA synthesis) at higher concentrations. In contrast to these conceivable mechanisms listed above, there is direct evidence of suppression of uranium respiration at >3.0 micromolar for a *Burkholderia* isolate from the Rifle study site [30]. *B*. *fungorum* strain Rifle uses U(VI) as a TEA for rRNA synthesis and cellular growth in a dose dependent manner. The rRNA synthesis data we present from the Rifle groundwater samples is consistent with this suppression of growth by uranium at concentrations above 4 micromolar. Therefore, given the observed differences in rRNA synthesis at high and low concentrations, respiration and growth on uranium is deemed the most likely mechanism by which the bacteria within our microcosms are generating ATP and synthesizing rRNA in a dose-dependent fashion from uranium addition.

Finally, this concept of sulfate-reducing microorganisms playing a role in uranium reduction in addition to *Geobacter*-like species at Rifle, CO has been articulated previously [31] and is supported by the chemical parameters and microbial community dynamics discerned from well D04 during the field additions (Figs 5 and 6). At the early stages of acetate addition (Day 7–12), uranium initially plummets as the *D*. *postgateii*-like TRF 215 begins to appear. *Desulfobacter postgateii* is a known sulfate reducing bacterium. If the cell numbers (16S gene dosage) are increasing, and sulfide is decreasing, another TEA must be utilized by this bacterium, such as uranium. However, by the onset of the groundwater flush (Day 15), TRF 215 has become a larger portion of the microbial community profile (30%) compared with TRF 212 (23%-Fig 6). This time point corresponds with the lowest uranium concentration measured in the field and the highest reduced iron levels (Fig 5). Since the other iron reducing bacteria are diminishing in the community profile and TRF 212 has increased, any reduced iron observed in the groundwater is likely a result of the *G*. *bemedjiensis*-like metabolic activity with respect to iron respiration. As the groundwater flush commences, uranium levels begin to increase (0.22 μM) and the acetate level drops. Yet, the uranium concentrations do not rebound to pre-acetate treatment levels (0.92 μM), indicating that uranium reduction continues in the field during this groundwater flush. By the end of the flush (Day 22), TRF 212 is replaced by TRF 213 (another *D*. *postgateii*-like OTU) and the uranium concentration drops to 0.16 μM. Sulfide actually drops during this second half of the flush from 9.1 to 3.8 μM, providing further evidence that TRF 213 and 215 are reducing terminal electron acceptors other than sulfate (such as uranium) in the field. As the acetate/bromide amendment is resumed, well D04 does not experience an increase in either solute until day 30, indicating subsurface flow by the amendment has by-passed this particular well. Interestingly, the microbial community completely resets to the day 12 profile.

In conclusion, both *Geobacter* and *Desulfobacter*-like microorganisms responded to the uranium amendments in replicate microcosm samples during the replicate years 2008 and 2009 as evidenced by the TRF 212, 213, and 215 peaks. There was no indication that *Burkholderia fungorum* strain Rifle was synthesizing rRNA in response to the uranium additions in our groundwater samples, which has a TRF size of 166 bp [30]. This result is consistent with a study of ^13^C-acetate incorporation in 2007 as field amendments commenced at this particular Rifle site [7]. Before acetate addition in the field, the TRF 166 was one of the major ^13^C labeled microorganisms in a SIP study using ^13^C-acetate in sediment microcosms. However, after field amendment of millimolar acetate, this particular TRF was neither active or detectable and *Geobacter*-like bacteria were dominant with respect to ^13^C-labeling.

These results suggest that *Desulfobacter*-like microorganisms may also be very important in the reduction of uranium in the field. Furthermore, there is proteome evidence indicating a legacy effect from the acetate in 2007 [32], leading to a primed sulfate-reducing community that was able to respond more quickly to the acetate in 2008 and 2009 [33, 34]. Given the high concentration of the acetate amendment, simultaneous iron, sulfate, and uranium reduction may have been happening in the field as has been measured in experimental columns [21]. In addition, *Desulfovibrio vulgaris* has been shown to be capable of reducing both sulfate and uranium in the same media and the presence of sulfate actually increased the uranium reduction rate [35]. The apparent increase in ribosomes from sulfate reducers occurred on a time frame of 15 days, when iron was readily available and the rate of sulfate reduction has not peaked. These findings suggest that both Fe- and sulfate reducers play a role in the *in-situ* removal of soluble uranium at the Rifle site after acetate field amendments.

## Supporting Information

S1 Fig Triplicate RT-PCR reactions using varying rRNA template mixtures (vol:vol). Scatter data are actual versus predicted TRF peak areas in relative fluorescent units (RFU) based on TRF peak area from original 1:1:1 mixture. Mix A 1:0.2:0.05 (closed diamond), Mix B 0.2:1:0.2 (open circle). The diagonal line represents the1:1 values.
(TIFF)

S2 Fig Part A. Example of nucleic acid extracts from Well D02 amended groundwater microcosms incubated for 24 h as described in the methods. Lanes are: A) Lambda HinD III molecular weight marker B) initial groundwater C) groundwater bottle incubation with no amendment D)—E)—F) 0.5 μM uranium addition + acetate G) 1.0 μM uranium addition + acetate H) 2.0 μM uranium addition + acetate I) 4.0 μM uranium addition + acetate J) 8.0 μM uranium addition + acetate K) 2.0 μM SO4 addition + acetate. Part B. Example of RT-TRFLP from a bottle incubation with no amendment (control), an acetate only amendment, and 2.0 μM uranium amendment + acetate for wells D02 and D07 from 2008.
(TIFF)

S3 Fig Additional RT-TRFLP controls for D02 and D07. (TIFF)

S4 Fig DNA-TRFLP profiles of 16S rRNA genes from the bacterial community (D04) on pre-filters collected during field amendment of acetate indicating days after injection begins (i.e. 7, 12, 22, 30 and 50; Wilkens *et al*., 2009). (TIFF)
