# Supplementary figures and images for: Identification of Bacteria Synthesizing Ribosomal RNA in Response to Uranium Addition During Biostimulation at the Rifle, CO Integrated Field Research Site
Source: PLoS One. 2015 Sep 18;10(9):e0137270. doi: 10.1371/journal.pone.0137270 (PMC4575074; doi:10.1371/journal.pone.0137270)

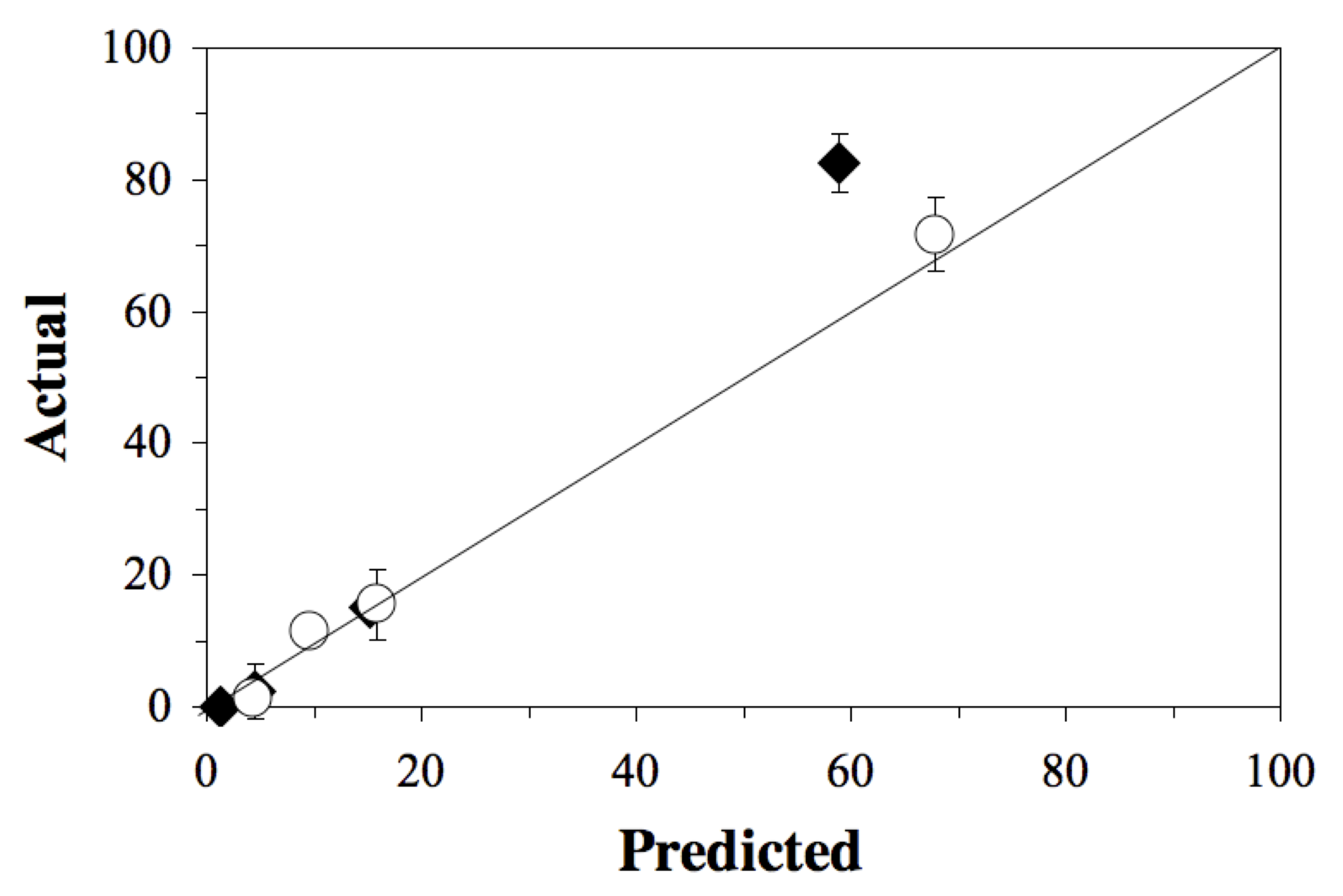

Supplement: S1 Fig — Scatter data are actual versus predicted TRF peak areas in relative fluorescent units (RFU) based on TRF peak area from original 1:1:1 mixture. Mix A 1:0.2:0.05 (closed diamond), Mix B 0.2:1:0.2 (open circle). The diagonal line represents the1:1 values. (TIFF) [file pone.0137270.s001.tiff]

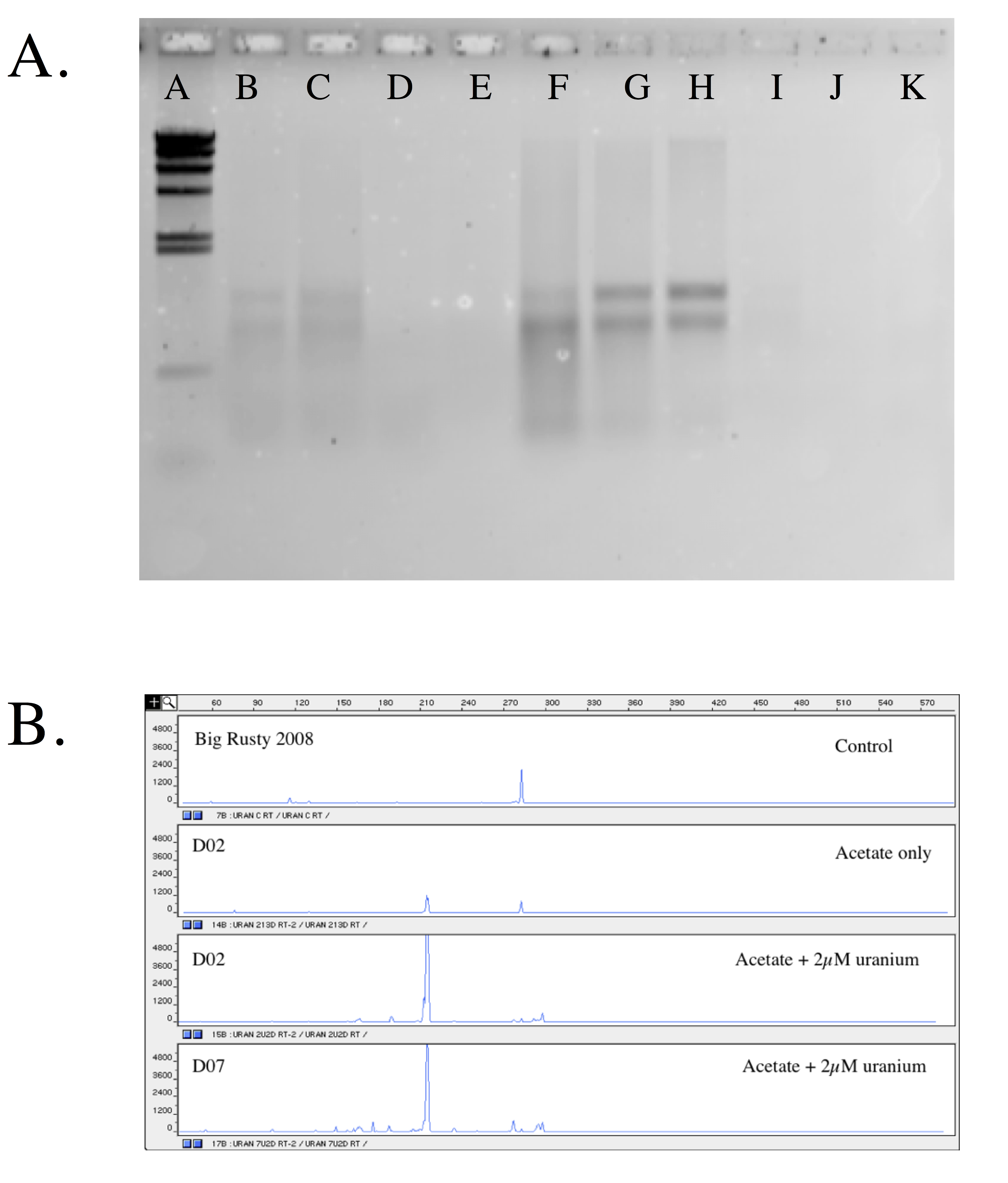

Supplement: S2 Fig — Part A. Example of nucleic acid extracts from Well D02 amended groundwater microcosms incubated for 24 h as described in the methods. Lanes are: A) Lambda HinD III molecular weight marker B) initial groundwater C) groundwater bottle incubation with no amendment D)—E)—F) 0.5 μM uranium addition + acetate G) 1.0 μM uranium addition + acetate H) 2.0 μM uranium addition + acetate I) 4.0 μM uranium addition + acetate J) 8.0 μM uranium addition + acetate K) 2.0 μM SO4 addition + acetate. Part B. Example of RT-TRFLP from a bottle incubation with no amendment (control), an acetate only amendment, and 2.0 μM uranium amendment + acetate for wells D02 and D07 from 2008. (TIFF) [file pone.0137270.s002.tiff]

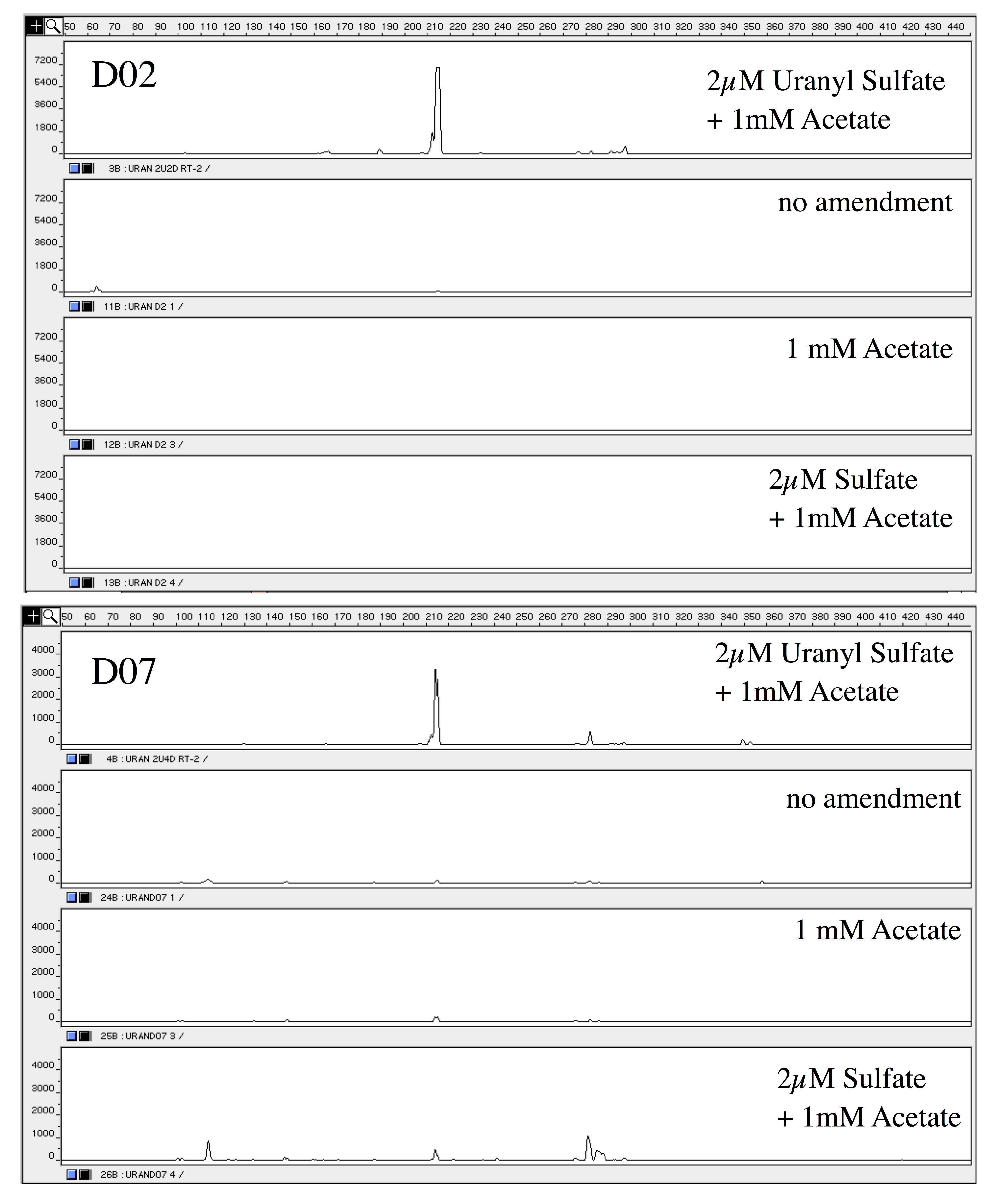

Supplement: S3 Fig — (TIFF) [file pone.0137270.s003.tiff]

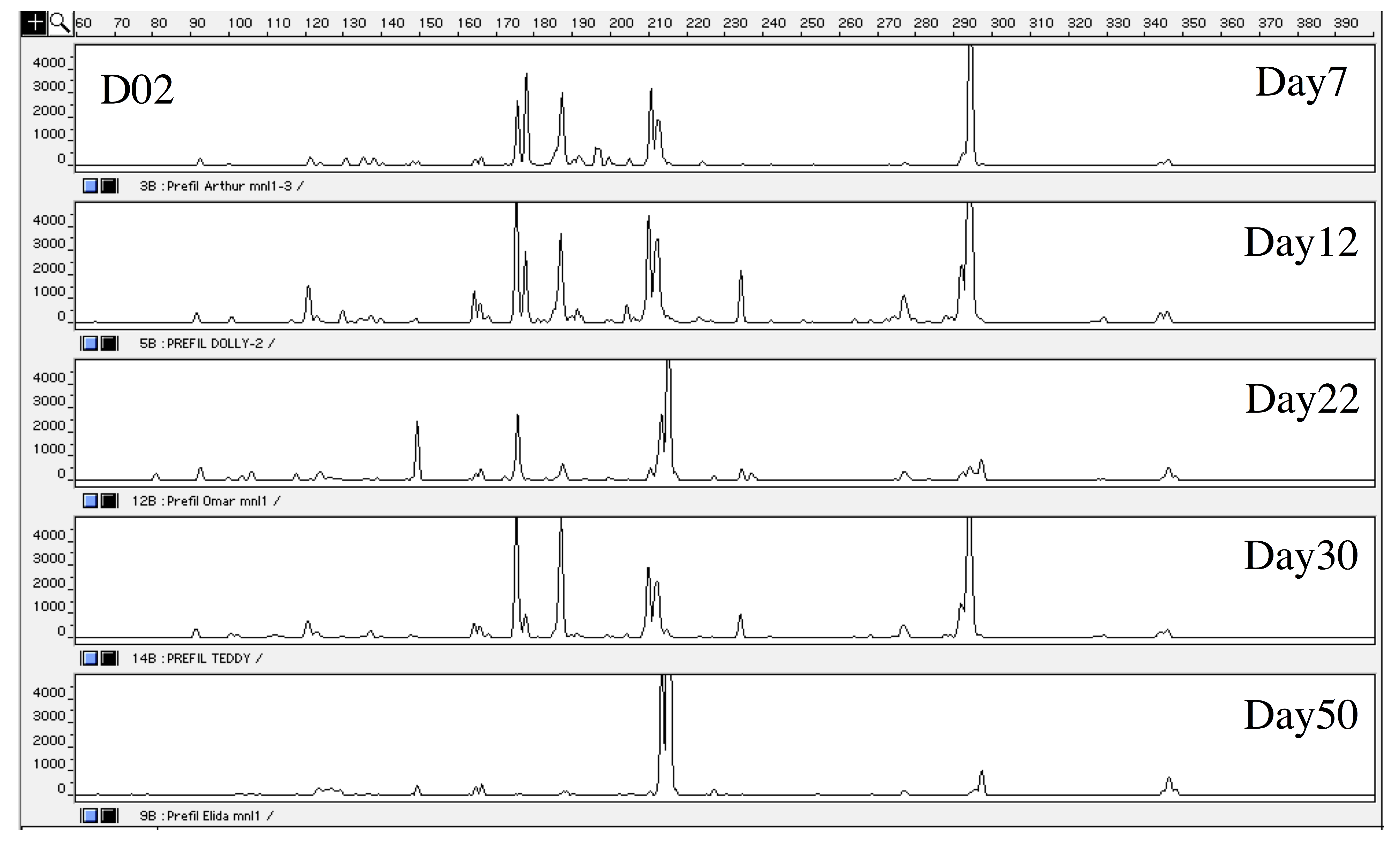

Supplement: S4 Fig — (TIFF) [file pone.0137270.s004.tiff]
